# CXC chemokine superfamily induced by Interferon-γ in asthma: a cross-sectional observational study

**DOI:** 10.1186/s40733-016-0021-y

**Published:** 2016-03-17

**Authors:** Yotaro Takaku, Tomoyuki Soma, Yoshitaka Uchida, Takehito Kobayashi, Kazuyuki Nakagome, Makoto Nagata

**Affiliations:** 1 Allergy Center, Saitama Medical University, Saitama, Japan; 2 Department of Respiratory Medicine, Saitama Cardiovascular and Respiratory Center, Saitama, Japan; 3 Department of Respiratory Medicine, Saitama Medical University, Saitama, Japan

**Keywords:** Asthma, Sputum, CXCR3 ligands, IP-10, Mig, Eosinophilic inflammation, Neutrophilic inflammation, Mixed granulocytic inflammation

## Abstract

**Background:**

Asthma is a disease encompassing a variety of contributing factors. Phenotyping of asthma based on the profile of accumulated granulocytes in the airways has been performed to explore the mediators involved in allergic bronchial inflammation. The aim of this study was to clarify the characteristics of the CXC chemokine superfamily induced by IFN-γ, namely CXCR3 ligands, in the airways of patients with asthma stratified by the differential proportion of granulocytes in sputum.

**Methods:**

Sputum was induced in 39 adult patients with asthma and 12 healthy subjects. Sputum samples were analyzed for total cell counts and differentials, and concentrations of IFN-γ–inducible protein 10 kDa (IP-10, CXCL10), monokine induced by IFN-γ (Mig, CXCL9), IFN-inducible T cell a chemoattractant (I-TAC, CXCL11), and IL-8 in the supernatants were assayed by ELISA.

**Results:**

Sputum concentrations of IP-10, Mig, and IL-8 were significantly higher in asthma than in healthy subjects. IP-10, Mig, and IL-8 were significantly higher in the mixed granulocyte subtype (eosinophils ≥ 2 % and neutrophils ≥ 40 % in sputum) than in healthy subjects. Additionally, IP-1 0 was significantly higher in the mixed granulocyte subtype than in eosinophil-predominant or neutrophil-predominant subtype (eosinophil percentage ≥ 2 % or neutrophil percentage ≥ 40 %). Mig and IL-8 were significantly higher in the mixed granulocyte subtype than in the paucigranulocyte subtype (eosinophils < 2 % and neutrophils < 40 % in sputum). I-TAC was not different between healthy subjects and asthmatics or granulocyte subtypes. All CXCR3 ligands were significantly associated with the composite of the eosinophil and neutrophil ratio in patients with asthma. Only Mig was significantly correlated with the total eosinophil and neutrophil ratio in patients with asthma on adjusted partial correlation analysis. Mig and IL-8 were significantly negatively correlated with forced expiratory volume in 1 s % predicted (% FEV_1_) in patients with asthma.

**Conclusions:**

CXCR3 ligands may serve as potent promoters in eosinophilic and neutrophilic airway inflammation in asthma.

**Electronic supplementary material:**

The online version of this article (doi:10.1186/s40733-016-0021-y) contains supplementary material, which is available to authorized users.

## Background

Asthma is a heterogeneous disease caused by genetic and environmental factors. The diversity of bronchial inflammation in asthma leads to difficulty treating severe asthma. To develop a better understanding of severe asthma, genotype, endotype, and phenotype cluster analyses of asthma have been used [1–8]. Recently, asthma endotype cluster analysis followed by genotype or phenotype cluster analysis has achieved successful clinical results, including anti-IL-5 antibody therapy for persistent eosinophilic severe asthma and anti- IL-4 or anti-IL-13 antibody therapy for high-Th2-type severe asthma [9–12]. These results imply that endotype cluster analysis is an important approach for determining the treatment of asthma.

There has been increasing evidence from endotype cluster analysis of asthma based on the kind of granulocyte that is predominant in the airway. Assessment on the basis of either the eosinophil or the neutrophil ratio has been performed [13–19]. In greater detail, cases are divided into 4 clusters for endotype cluster analysis of asthma, including the paucigranulocytic inflammatory subtype, the eosinophil-predominant inflammatory subtype, the neutrophil-predominant inflammatory subtype, and the mixed granulocyte inflammatory subtype [20]. Two Severe Asthma Research Program (SARP) studies reported that moderate-to-severe asthma was characterized by neutrophil-predominant or mixed granulocytic inflammation [21, 22]. Adult-onset severe asthma has higher sputum eosinophil and blood neutrophil counts than mild-to-moderate persistent asthma [23]. The stratification of asthma based on the predominant granulocyte in sputum has also shown elevation of some cytokines or chemokines [21–28].

IFN-γ–inducible protein 10 kDa (IP-10, CXCL10), monokine induced by IFN-γ (Mig, CXCL9), and IFN-inducible T cell a chemoattractant (I-TAC, CXCL11) belong to the CXC chemokine subfamily that binds to CXCR3 [29, 30]. CXCR3 ligands have been considered to be involved in the Th1-type immune response because they are produced by various cells in response to IFN-γ [29, 30]. In general, CXCR3 ligands accumulate mainly in lymphocytes and subsequent neutrophils. The expression of CXCR3 ligands has been shown to be up-regulated in delayed-type immune responses of the skin, in murine macrophages infected with *Mycobacterium tuberculosis* [31], atherosclerosis [32], viral meningitis [33], and viral-activated neutrophils [34]. Mig has been shown to be increased in bronchoalveolor lavage (BAL) fluids obtained from patients with acute respiratory distress syndrome in cooperation with CXC3 receptor expression on neutrophils in BAL fluid [34].

Recently, IP-10 has been reported to be involved in allergic bronchial inflammation. For example, IP-10 in BAL increased to biologically relevant levels in patients with asthma after segmental allergen challenge [35]. Serum IP-10 concentration was specifically increased in rhinovirus-induced asthma exacerbation [36]. However, the available information in terms of the contribution of IP-10 to asthma exacerbation remains insufficient to determine whether IP-10 and other CXCR3 ligands are also implicated in allergic bronchial inflammation in stable asthma. Moreover, whether trends in CXCR3 ligands depend on the severity of asthma or on granulocytic inflammatory subtype in asthma has never been fully studied.

We hypothesized that CXCR3 ligands would be elevated in stable asthma and would characterize granulocytic inflammatory subtypes in asthma. To clarify this, the concentrations of CXCR3 ligands and IL-8 were measured in sputum obtained from patients with asthma and healthy volunteers. The differences in these chemokines between the grades of asthma severity according to the European Respiratory Society (ERS)/American Thoracic Society (ATS) statement and their associations with respiratory functions were investigated [37]. Finally, the levels of CXCR3 ligands were compared among granulocytic inflammatory asthma subtypes, especially mixed granulocyte inflammatory subtype.

## Methods

### Patients

Patients with asthma and normal control subjects were recruited from the Allergy Center of the Saitama Medical University Hospital. Asthma was defined according to the GINA guidelines [38], including a clear clinical history of current symptoms and either an increase in baseline forced expiratory volume in 1 s (FEV_1_) of ≥12 % over baseline values after inhalation of 200 μg of salbutamol aerosol, or the presence of bronchial hyperresponsiveness defined by methacholine PC20 of < 4 mg/mL. Exclusion criteria were: (1) lung disease other than asthma considered to interfere with the evaluation; (2) admission to an emergency room/intensive care unit because of asthma during the previous 1 month; (3) 3 or more courses of oral corticosteroids or hospitalization for asthma during the previous 1 month; (4) respiratory infections during the previous 1 month; (5) history of being treated with immunosuppressants; (6) pregnant; and (7) comorbid systemic diseases, including cancer, severe renal failure, or severe heart failure; and (8) current smoker. Healthy subjects were recruited from the hospital staff and had no history of asthma or other respiratory disease and FEV_1_ > 80 % predicted. All subjects performed pulmonary function tests, and forced vital capacity (FVC) and FEV_1_ were measured according to the ATS guidelines [39] using an AS307 spirometer (Minato Medical Science, Osaka, Japan). The fraction of exhaled nitric oxide (FeNO) was also measured for all subjects using a chemiluminescence analyzer (SiEVER 280i NIPPON MEGACARE Co, Ltd, Tokyo, Japan) with a resolution of 1 part per billion (ppb) according to the recommendations of the ATS. Severe asthma was defined according to the International ERS/ATS guidelines published in 2014 [37], in which severe asthma required treatment with high-dose inhaled corticosteroid (ICS) and second controllers, since guidelines suggested medications for Global Initiative For Asthma (GINA) steps 4–5 asthma for the previous year or systemic corticosteroid for ≥ 50 % of the previous year to prevent it from becoming “uncontrolled” or which remains “uncontrolled” despite this therapy. The study was approved by the Institutional Review Board of the Saitama Medical University Hospital. Written informed consent was obtained from all individuals.

### Sputum induction and processing

Induced sputum was collected as described previously [40–42]. Salbutamol was administered using a metered dose inhaler. Fifteen minutes later, sterile hypertonic saline (4.5 %) was inhaled using an ultrasonic nebulizer at room temperature. Sputum was collected at 5-min intervals for up to 30 min. Subjects were asked to rinse their mouth with water before each expectoration to minimize salivary contamination. All initial samples were discarded. Induced sputum samples collected into 50-mL polypropylene tubes were stored at 4 °C for further processing. Hanks’ balanced salt solution (HBSS; 1 mL) containing 1 % dithiothreitol (Sigma, St. Louis, MO, USA) was added to sputum samples, and they were gently vortex-mixed and repeatedly aspirated at ambient temperature until the mixture was homogeneous. Samples were diluted with HBSS to 5 mL, and separated by centrifugation at 400 g for 10 min. Cytospin slides were prepared by cytospin (Cytospin 3: Shandon, Tokyo, Japan) and stained by May-Grunwald-Giemsa for differential cell counts. At least 500 inflammatory cells were counted for each sample by an independent investigator. Cytospin slides with squamous epithelial cells accounting for 50 % or less were deemed adequate for analysis. Concentrations of IP-10, Mig, and I-TAC in sputum supernatants were measured using a specific enzyme immunoassay (Cayman Chemicals; Ann Arbor, MI). The lower limit of detection for these assays was 31.2 pg/mL for IP-10, 62.5 pg/mL for Mig, and 7.81 pg/ml for I-TAC. When any value was lower than the lower limit or not detected, the lower limit of values were assigned to subjects.

### Subject classification in accordance with sputum eosinophil percent and neutrophil percent

Subjects were categorized into 3 subgroups based on the percentages of eosinophils and neutrophils in their sputum; the paucigranulocytic subtype (eosinophil percentage < 2 % and neutrophil percentage < 40 %), the eosinophil-predominant or neutrophil-predominant subtype (eosinophil percentage ≥ 2 % or neutrophil percentage ≥ 40 %), and the mixed granulocyte subtype (eosinophil percentage ≥ 2 % and neutrophil percentage ≥ 40 %). Various cut-off points of sputum eosinophil (1 to 3 %) and neutrophil percentages (40 to 64 %) have been applied to the classification of asthma inflammatory subtype [20–22, 25]. In the SARP studies, eosinophil percentage ≥ 2 % and neutrophil percentage ≥ 40 % in sputum were chosen for dividing the subjects into the paucigranulocytic subtype, the eosinophil-predominant subtype, the neutrophil-predominant subtype, and the mixed granulocyte subtype [21, 22], because classifying severe asthma patients at 61 % of sputum neutrophil percentage, which corresponded to the 95th percentile of sputum neutrophil percentages in healthy control subjects and was adopted as a cut-off point for neutrophil percentage in an earlier study [20], allocated 75 % of the patients with severe asthma to the non-neutrophilic asthma subtype. Similar to the SARP studies, this point was considered too strict a distinction, since only 13 % (4/31) of all asthma patients were placed in neutrophilic categories in our study. Thus, 2 % for the eosinophil percentage and 40 % for the neutrophil percentage were considered to be the appropriate cut points for classifying the cases into 3 subgroups.

### Statistical analysis

Values are expressed as means ± standard deviation (SD) or medians with 25th–75th percentile ranges if not normally distributed. The Kolmogorov-Smirnov test or the Leven test was performed to confirm a normal distribution. Parametric continuous variables in two groups were tested by Student’s *t-*test. Nonparametric variables in two groups were tested by the Mann–Whitney U test and in more than two groups by the Kruskal-Wallis test. Initial analyses with a significant difference were further explored by post hoc pairwise analyses (Bonferroni correction). Associations between data were determined using Pearson correlation coefficients. Categorical variables were analyzed using χ^2^ tests. Values of *P* < 0.05 were considered significant. Data were analyzed using SPSS version 20.0 (IBM, Armonk, New York, USA).

## Results

### Subjects’ demographics and characteristics

Forty eight patients with asthma and 19 normal control subjects were enrolled. Appropriate sputum samples were obtained from 39 asthmatic patients (80 %) and 12 normal control subjects (63 %). Twenty eight patients (72 %) had non-severe asthma, and 11 patients (28 %) had severe asthma. The demographics and clinical characteristics of the subjects in these groups are shown in Table 1 and Table S1 (see Additional file 1). An additional data file shows this in more detail. There were no significant differences in BMI and the number of patients with atopy among the groups. Patients with asthma, particularly with severe asthma were older than healthy subjects. The percentage of female and ex-smokers in patients with asthma was significantly higher than in healthy subjects. The duration of disease of patients with severe asthma was significantly longer than of non-severe asthma. Patients with severe asthma were treated with significantly higher doses of ICS than those with non-severe asthma. The percentage of severe asthma treated with oral corticosteroids (OCS) and leukotriene receptor antagonist was higher than non-severe asthma. FEV_1_ and % FEV_1_ were significantly lower in patients with non-severe and severe asthma than in healthy subjects (*p* < 0.0001).

**Table 1:** Characteristics of Subjects

	Asthma patients	Healthy subjects	*P* value*
Subjects, n (%)	39	12	
Age, y	55 (20–72)	47 (30–57)	0.02
Male/female, n (Male, %)	16 (41)/23	4 (33)/9	0.05
BMI (kg/m^2^)	22.5 (3.8)	22.2 (3.1)	N.S.
Duration of asthma (y)	6.5 (2–16.5)	N. A.	N. A.
Ex-smokers, n (%)	14 (36)	0 (0)	0.02
Medication			
ICS dose (μg/day)	995 (400–1300)	N. A.	N. A.
Long acting β2-agonist, n (%)	27 (69)	N. A.	N. A.
Leukotriene receptor antagonist, n (%)	27 (69)	N. A.	N. A.
Oral corticosteroids, n (%)	8 (21)	N. A.	N. A.
Atopy, n (%)	25 (64)	5 (42)	N.S.
Asthma control and health care use, past year			
β-agonist use/day	0.00 (0.00–0.03)	N. A.	N. A.
Oral corticosteroid bursts	1.0 (0.0–2.0)	N. A.	N. A.
FEV1, L/min	2.0 (0.4)	3.2 (0.8)	<0.0001
FEV1, % predicted	80.4 (20.3)	105.5 (14.6)	<0.0001
FeNO, ppb	29.5 (14.0–45.5)	N.D.	N. A.
Cell population in sputum			
% Squamous	16.7 (7.6–25.2)	32.9 (25.1–42.4)	0.03
% Macrophage	26.0 (19.0–43.1)	37.5 (27.6–4 3.2)	N. S.
% Lymphocyte	7.3 (4.6–11.9)	7.5 (3.7–13.4)	N. S.
% Neutrophil	29.9 (24.1–51.2)	21.6 (18.6–24.1)	N. S.
% Eosinophil	6.4 (1.6–11.4)	0.5 (0.0–2.0)	<0.0001
% Eosinophil	38.3 (29.8–63.1)	23.9 (18.6–24.8)	0.002
+ % Neutrophil			
CXC chemokine in sputum			
IP-10 (pg/mL)	60.1 (31.2–306.7)	31.2 (0.0)	0.01
Mig (pg/mL)	989.5 (62.5–2698.2)	62.5 (0.0)	<0.0001
I-TAC (pg/mL)	7.8 (7.8–24.3)	7.8 (0.0)	N. S.
IL-8 ( Log, pg/mL)	3120.3 (3.5, 1442.9 - 9115.1)	1356.6 (3.0, 522.6 - 2550.4)	0.01

Data are presented as means (SD) unless otherwise indicated. Nonparametric data are shown as medians (25 to 75 %). Age is shown as mean (minimum to maximum)

ICS = inhalation corticosteroid (beclomethasone), where 1 μg beclomethasone = 1 μg budesonide = 0.5 μg fluticasone

*P values are calculated with the use of the Mann–Whitney U test for nonparametric data, Student’s *t-*test for variables with a parametric distribution, and Fisher’s exact test for comparison of proportions

N. A.; not applicable. N.D.: not done

### Sputum inflammatory cell pattern based on the classification by asthma severity

Eosinophils ratio was higher in patients with asthma, especially with severe asthma than in healthy subjects (Table 1). The total ratios of eosinophils and neutorphils in sputum were higher in patients with severe and non-severe asthma than in healthy subjects (see Additional file 2: Table S2). The prevalence of patients with severe asthma was higher in the eosinophil-predominant subtype (33 %) and the mixed granulocytic subtype (25 %) than in the paucigranulocytic subtype (20 %). There was a significant negative correlation between the total eosinophil ratio and neutrophil ratio in sputum and % FEV_1_ in all subjects (*r* = −0.38, *p* = 0.009).

### CXC chemokines in sputum based on the classification by asthma severity

**Fig. 1:**
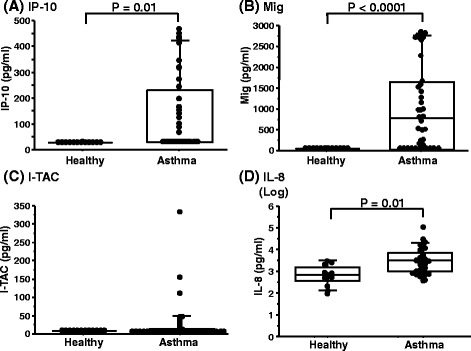
Sputum CXCR3 ligands and IL-8 in patients with asthma and healthy subjects. The comparison of sputum CXCR3 ligands and IL-8 between in patients with asthma and healthy subjects is shown in **a** IP-10, **b** Mig, **c** I-TAC and **d** IL-8. Each closed dot represents a different subject. Boxes represent the 25th and 75th percentiles, the central line represents the median, and solid circles represent outliers. Error bars indicate SDs. The comparison of IP-10 and I-TAC between groups was analyzed using the Mann–Whitney U test. The comparison of Mig and IL-8 was analyzed using Student’s *t-*test

IP-10, Mig, and I-TAC were not detected in sputum of any healthy subjects. In 17, 11 and 28 of patients with asthma, IP-10, Mig and I-TAC were below the detection limit in sputum, respectively. IP-10, Mig and IL-8 levels, but not I-TAC levels, were higher in asthmatic patients than in healthy subjects (Fig. 1). There was no difference in any CXC chemokine between non-severe asthma and severe asthma patients (Additional file 2: Table S2).

### Subjects’ demographics and other characteristics based on the classification by percentages of eosinophils and neutrophils in sputum

The proportion of patients with severe asthma was high in the mixed granulocytic subtype and in the eosinophil-predominant subtype, similar to the result in the study from the SARP group [22, 23]. Kaminska et al. showed that the total eosinophil and neutrophil ratio in sputum was associated with airway obstruction [43]. Additionally, eosinophil and neutrophils were shown to express CXC3 receptors [44, 45]. Therefore, we decided to examine the patterns of CXC chemokines according to the stratification based on the percentages of eosinophils and neutrophils in the sputum.

The paucigranulocytic subtype (*n* = 9), the eosinophil or neutrophil-predominant subtype (*n* = 22), and the mixed granulocytic subtype (*n* = 8) were analyzed. The eosinophil or neutrophil-predominant subtype was composed of the eosinophil-predominant subtype (*n* = 19) and the neutrophil-predominant subtype (*n* = 3). The demographics and other characteristics of them are shown in Table 2. There were no significant differences in age, sex, BMI and number of patients with atopy among the 4 groups. There were no significant differences in dose of ICS, daily OCS use, adjunctive therapy and frequency of rescue β2-agonist use or asthma exacerbation with OCS treatment in the past year among the 3 granulocytic subtypes. The duration of disease of patients with the mixed granulocytic subtype was significantly longer than with the paucigranulocytic subtype. Patients in the eosinophil or neutrophil-predominant subtype and the mixed granulocytic subtype had significantly lower FEV_1_ and % FEV_1_ than healthy subjects. There were no differences in pulmonary functions and FeNO among the 3 granulocytic asthma subtypes.

**Table 2:** Characteristics of subjects stratified by percentage of eosinophils and neutrophils in sputum

	Paucigranulocytic	Eosinophil-predominant or Neutrophil-predominant	Mixed granulocytic	Healthy Subjects	Overall
					*P* value*
Subjects, n (%)	9 (23)	22 (56)	8 (21)	12	
Eos dominant/Neu dominant, n (%)	N. A.	19/3 (86/14)	N. A.	N. A.	
Age, y	50 (20–71)	58 (30–71)	55 (22–72)	47 (30–57)	N. S.
Male/female, n (Male, %)	3 (33)/6	9 (41)/13	4 (50)/4	4 (33)/8	N. S.
BMI	24.5 (6.1)	21.8 (2.5)	21.9 (1.0)	22.1 (3.1)	N. S.
Duration of asthma (y)	5.1 (5.6)	10.8 (9.7)	23.4 (20.5)†	N. A.	0.01
Ex-smokers, n (%)	4 (44)	9 (41)	1 (12)	0 (0)	0.03
Non-severe/Severe, n (Severe, %)	6/1 (11)	14/8 (36)	6/2 (25)	N. A.	N. S.
Medication					
ICS dose (μg/day)	900 (500–1150)	800 (400–1150)	800 (400–1200)	N. A.	N. S.
LABA, n (%)	6 (67)	12 (55)	7 (88)	N. A.	N. S.
LTRA/TP, n (%)	6 (67)/3 (33)	13 (59)/11 (50)	7 (88)/4 (50)	N. A.	N. S.
Oral corticosteroids, n (%)	0 (0)	4 (18)	1 (13)	N. A.	N. S.
Atopy, n (%)	5 (57)	14 (64)	6 (75)	5 (42)	N. S.
Asthma control and health care use, past year					
β-agonist use/day	0.00 (0.00–0.01)	0.00 (0.00–0.02)	0.00 (0.00–0.68)	N. A.	N. S.
Oral Corticosteroid bursts	1.0 (1.0–2.5)	1.0 (0.0–1.0)	1.0 (0.0–2.0)	N. A.	N. S.
FEV_1_, L/min	2.3 (0.6)	2.1 (0.9)‡	2.0 (1.0)§	3.2 (0.8)	0.05
FEV_1_, % predicted	89.7 (14.9)	81.3 (22.4)װ	72.9 (18.2)¶	105.5 (14.6)	0.01
FeNO, ppb	18.5 (12.3–45.5)	34.5 (13.3–51.0)	53.2 (21.5–99.0)	N.D.	N. S.

Data are presented as means (SD) unless otherwise indicated. Nonparametric data are shown as medians (25 to 75 %). Age is shown as mean (minimum to maximum)

ICS = inhalation corticosteroid (beclomethasone), where 1 μg beclomethasone = 1 μg budesonide = 0.5 μg fluticasone

*P values are calculated with the use of the Kruskal-Wallis test and the Mann–Whitney U test for nonparametric data, ANOVA for variables with a parametric distribution, and Fisher’s exact test for comparison of proportions. Initial analyses with a significant difference are further explored by post hoc pairwise analyses (Bonferroni correction)

LABA, Long acting β2-agonist; LTRA, Leukotriene receptor antagonist; Eos, eosinophil, Neu, neutrophil

vs. Paucigranulocytic, † *p* = 0.01

vs. Healthy, ‡ *p* = 0.006; § *p* = 0.02; װ *p* = 0.004; ¶ *p* = 0.002

N. A.; not applicable. N.D.: not done

### CXC chemokines in sputum based on the sputum inflammatory granulocytic pattern

**Fig. 2:**
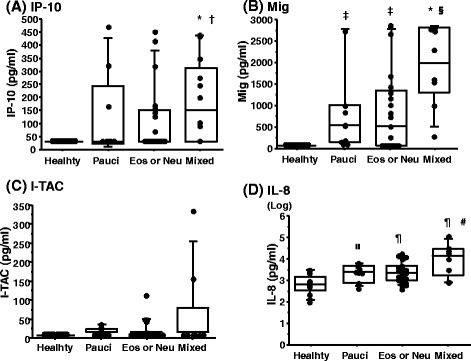
Sputum CXCR3 ligands and IL-8 in asthmatic patients classified by the sputum granulocyte ratio. The comparison of sputum CXCR3 ligands and IL-8 among the 3 granulocytic subtypes and healthy subjects is shown in **a** IP-10, **b** Mig, **c** I-TAC and **d** IL-8. Each closed dot represents a different subject. Boxes represent the 25th and 75th percentiles, the central line represents the median, and solid circles represent outliers. Error bars indicate SDs. The comparison of CXCR3 ligands and IL-8 was analyzed using the Kruskal-Wallis test and the Mann–Whitney U test. Initial analyses with a significant difference were further explored by post hoc pairwise analyses (Bonferroni correction). * v. s. Healthy, *p* < 0.0001; † v. s. Eos or Neu, *p* = 0.04; ‡ v. s. Healthy, *p* = 0.04; § v. s. Pauci, *p* = 0.008; װ v. s. Healthy, *p* = 0.05; ¶ v. s. Healthy, *p* = 0.002; # v. s. Pauci, *p* = 0.05. Pauci, Paucigranulocytic subtype; Eos or Neu, Eosinophil-dominant or Neutrophil-dominant subtype; Mixed, Mixed subtype

To elucidate whether sputum CXC chemokines differed among the 3 granulocytic asthma subtypes, CXC chemokine data were evaluated according to the classification by sputum inflammatory granulocytic pattern (Fig. 2, Table 3). IP-10 was significantly higher only in the mixed granulocytic subtype than in the eosinophil or neutrophil-predominant subtype and in healthy subjects (overall *P* = 0.002, Fig. 2a). Mig and IL-8 were significantly higher in all 3 granulocytic subtypes than in healthy subjects (overall *P* < 0.0001, 0.04, respectively, Fig. 2b and d). Mig and IL-8 were also significantly higher in the mixed granulocytic subtype than in the paucigranulocytic subtype. I-TAC was not different among the groups (Fig. 2c).

**Table 3:** Sputum cell characteristics, CXC3R ligands, and IL-8 stratified by subjects’ sputum eosinophil and neutrophil percentages

	Paucigranulocytic	Eosinophil-predominant or Neutrophil-predominant	Mixed granulocytic	Healthy subjects	Overall
					*P* value*
Subjects, n	9	22 (Eos/Neu, 19/3 )	8	12	
Cell population in sputum					
% Squamous	30.5 (14.1)	18.5 (11.8)	12.2 (8.0) †, ‡	30.4 (11.3)	0.002
% Mac	40.2 (12.1) †, §	28.9 (13.1)	15.5 (8.0)	19.6 (19.7)	0.002
% Lym	4.7 (4.2)	9.7 (6.3)	4.1 (2.6)	6.3 (7.7)	N. S.
% Neu	23.7 (9.7)	30.4 (17.6)	57.1 (10.7) װ, ¶, #	32.6 (26.9)	0.003
% Eos	1.0 (0.6)	13.9 (14.5) ††, ‡‡	10.9 (11.5)	0.5 (1.0)	0.002
% Eos + % Neu	24.6 (10.2)	45.1 (19.0) †, ‡	68.1 (10.5)§§, װװ, ¶¶	21.6 (5.5)	<0.0001
CXC chemokine in sputum					
IP-10 (pg/mL)	31.2 (31.2–242.1)	31.2 (31.2–156.6)	221.5 (92.7–411.6) §§,¶¶	31.2 (31.2–31.2)	0.002
Mig (pg/mL)	588.1 (105.1–902.6) ##	763.3 (62.5–1916.0) ##	1938.1 (1121.1–2757.7) §§, †††	62.5 (62.5–62.5)	<0.0001
I-TAC (pg/mL)	7.8 (7.8–26.6)	7.8 (7.8–16.2)	7.8 (7.8–244.3)	7.8 (7.8–7.8)	N. S.
IL-8 (Log, pg/mL)	2336.4 (3.2, 697.1–3734.5) ‡‡‡	4079.4 (3.4, 957.7–4947.5) §§§	13305.5 (3.9, 1373.5–28075.2) §§§, װװװ	622.0 (2.9, 276.3–1709.6)	0.04

Data are presented as means (SD) unless otherwise indicated. Nonparametric data are shown as medians (25 to75 %)

*P values are calculated with the Kruskal-Wallis test and the Mann–Whitney U test for nonparametric data and ANOVA for variables with a parametric distribution. Initial analyses with a significant difference are further explored by post hoc pairwise analyses (Bonferroni correction). Mac, Macrophages; Lym, Lymphocytes; Eos, eosinophils; Neu, neutrophils

v. s. Healthy,† *p* = 0.01; װ *p* = 0.03; †† *p* = 0.006; §§ *p* < 0.0001; ## *p* = 0.04; ‡‡‡ *p* = 0.05; §§§ *p* = 0.002

v. s. Pauci, ‡ *p* = 0.01; ¶ *p* = 0.003; ‡‡ *p* = 0.02; װװ *p* < 0.0001; ††† *p* = 0.008; װװװ *p* = 0.05

v. s. Eosinophil-predominant or Neutrophil-predominant, # *p* = 0.006; ¶¶ *p* = 0.04

v. s. Mixed, § *p* = 0.005

**Fig. 3:**
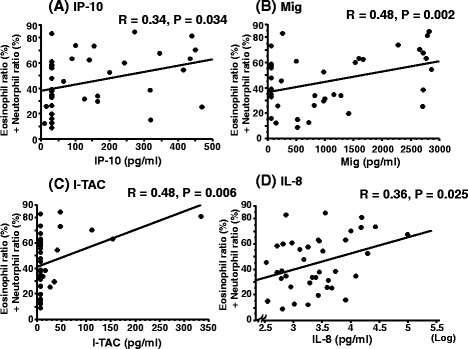
Correlations between CXCR3 ligands or IL-8, and total sputum eosinophil and neutrophil percentages in asthmatic patients. Associations between data were determined using Pearson correlation coefficients. Correlations between combination of eosinophil percentage and neutrophil percentage in sputum and **a** IP-10, **b** Mig, **c** I-TAC and **d** IL-8 in sputum

IP-10, Mig, I-TAC and IL-8 in sputum were significantly correlated with the total sputum eosinophil and neutrophil ratio in patients with asthma (Fig. 3a, b, c and d). IP-10 in sputum had no relationships with the ratios of either eosinophils or neutrophils in sputum in patients with asthma. Mig, I-TAC and IL-8 in sputum were significantly correlated with the sputum neutrophil ratio, but not eosinophil in patients with asthma (neutrophil ratio v. s. Mig, *R* = 0.32, *P* = 0.05; I-TAC, *R* = 0.45, *P* = 0.01; IL-8, *R* = 0.40, *P* = 0.02). IP-10 and Mig were significantly correlated with IL-8 in patients with asthma (IP-10, *R* = 0.42, *P* = 0.007; Mig, *R* = 0.64, *P* < 0.0001). The partial correlation analysis adjusted with age, eosinophil ratio and neutrophil ratio in sputum revealed that only Mig was significantly correlated with the total of eosinophil ratio and neutrophil ratio (*R* = 0.40, *P* = 0.035) in patients with asthma.

**Fig. 4:**
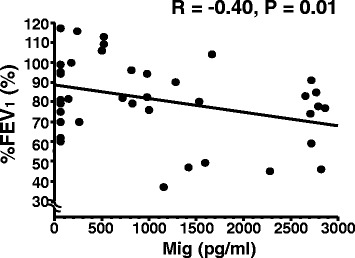
Correlations between sputum CXCR3 ligands, and FEV_1_ % predicted in asthmatic patients. Associations between data were determined using Pearson correlation coefficients. Correlations between Mig in sputum and FEV_1_ % predicted is shown

Mig was significantly negatively correlated with % FEV_1_ in patients with asthma (Fig. 4). IL-8 was also negatively correlated with % FEV_1_ in patients with asthma (*R* = −0.47, *P* = 0.002). The adjusted partial correlation analysis revealed that Mig and IL-8 were significantly negatively correlated with % FEV_1_ (Mig, *R* = −0.34, *P* = 0.039; IL-8, *R* = −0.47, *P* = 0.003) in patients with asthma. These negative correlations between them were detected in all subjects with the adjusted partial correlation analysis (Mig, *R* = −0.40, *P* = 0.005; IL-8, *R* = −0.62, *P* < 0.0001). Neither in patients with asthma nor all subjects, IP-10 and I-TAC were associated with pulmonary function.

## Discussion

This study showed that sputum IP-10 and Mig, members of the CXCR3 ligands family, and IL-8 were higher in asthma patients than in healthy subjects. Additionally, according to the classification based on the proportion of inflammatory granulocytes in sputum, IP-10, Mig and IL-8 in sputum were higher in the mixed granulocyte subtype than in healthy subjects. Elevations of Mig and IL-8 were specifically intensified in the mixed granulocyte subtype compared with the other granulocytic distribution asthma subtypes. Nevertheless, the differences in CXC chemokines between the severities of asthma were marginal. These results suggest that CXC chemokines are more relevant to express the characteristics of asthma endotypes based on the proportion of sputum inflammatory granulocytes than asthma phenotypes based on the clinical severities of asthma. Furthermore, three CXCR3 ligands were associated with the total eosinophil and neutrophil ratio in patients with asthma. Mig was also associated with pulmonary function in patients with asthma. Consequently, CXCR3 ligands may be involved in accumulated inflammatory granulocytes in the airways, and Mig may be involved in the progression of the pathophysiology in the airway in asthma. CXCR3 ligands might serve as a relevant marker of the mixed granulocyte asthma subtype.

Viral infection- or allergen-triggered exacerbation of allergic airway inflammation has been shown to increase IP-10 in serum or BAL [35, 36]. The present findings demonstrated the new information that IP-10 and Mig were similarly elevated in sputum even in stable asthma patients. CXCR3 is not exclusive to Th1 cells although engagement of CXCR3 in Th1-type lymphocytes has been the subject of particular interest. When activated, Th2 cells express low levels of CXCR3 [46] and they seem to be selectively recruited to the airways in response to CXCR3 ligands [47, 48]. Chronic asthmatic patients with high expressions of IL-27 mRNA on BAL cells and of CCL26 mRNA on epithelial cells, which can be interpreted as evidence of high Th1 and high Th2 immunity, showed increased Mig mRNA expression on epithelial cells [49]. Diverse environmental factors that promote the Th1 immune axis, such as a workplace agent [50], atypical bacteria [51], or fungi [52], may help continuous production of CXCR3 ligands, since they are produced by residual cells and leukocytes in response to toll-like receptors or interferons [53]. The production of CXCR3 ligands has been known to be induced by interferons. Therefore, IP-10 and Mig are likely involved in the pathogenesis of chronic asthma.

In this study, sputum IP-10 and Mig levels in asthma differed among the 3 granulocytic subtypes while not between the clinical asthma severities. Additionally, CXCR3 ligands were positively related to the total proportion of eosinophils and neutrophils in asthma. These results suggests that sputum CXCR3 ligand levels in asthma seemed to represent the asthma phenotype based on the ratios of granulocytes in sputum, rather than the clinical asthma severity. Several studies showed similar results. Eosinophilic asthma or asthma naïve to corticosteroids has higher expressions of eotaxin mRNA and proteins in bronchial tissue or sputum cells [27, 28, 54]. Neutrophilic asthma has increased IL-8 in sputum [24, 25]. The SARP group’s report showed that brain-derived neurotrophic factor, IL-1β, and macrophage inflammatory protein 3a/CCL20 in sputum increased in asthmatic patients with sputum containing ≥40 % neutrophils and tended to be higher in those who with ≥40 % neutrophils and ≥2 % eosinophils [21]. Considering these evidences, there were some chemokines engaged in specific granulocyte recruitment to the airway, leading to eosinophil or neutrophil-predominant asthma.

Mig appeared to exert more specific roles than the other CXCR3 ligands in the mixed granulocyte subtype in the present study. Mig was markedly increased in the mixed granulocyte subtype compared with the other subtypes, whereas IP-10 was moderately elevated. Furthermore, the partial correlation analysis adjusted with age, eosinophils and neutrophils, revealed that only the association between Mig and elevations of both granulocytes was preserved in asthmatic patients. Mig was also associated with deterioration of % FEV_1_ in asthmatic patients. These findings support the findings in the study reported by Xie et al. They found that Mig-expressing epithelial cells increased in asthmatic patients with high expressions of Th1-type cytokine mRNA on BAL cells and of Th2-type chemokine mRNA on epithelial cells, and that those patients had the lowest % FEV_1_ among the other asthma subtypes including Th1 cytokine high expression/Th2 chemokine low expression subtype, Th1 low/Th2 high, or Th1 low/ Th2 low [49]. Therefore, Mig may represent the pathophysiologic features of the mixed granulocyte subtype (high Th1 and high Th2 response).

The elevations of IP-10, Mig, and IL-8 in the mixed granulocyte subtype would be attributed to the predominant granulocyte of the two elevated granulocytes. Neutrophils were relatively predominant in the mixed granulocyte subtype in the present study. Recent studies have shown the expression of CXCR3 on neutrophils from patients with chronic lung disease, but not in healthy control subjects [45]. CXCR3 was highly expressed in inflamed neutrophils in BAL obtained from influenza virus-infected mice, and a reduction of neutrophils in BAL was reported in CXCR3 or IP-10 knockout mice with viral- or acid-induced acute respiratory distress syndrome [34]. Therefore, IP-10 and Mig may promote neutrophil accumulation in the airways. Likewise, the increase of eosinophils in sputum may affect this phenomenon. In vitro, our previous study showed that IP-10 and Mig enhanced adhesiveness of eosinophils to ICAM-1 [55], which may be associated with elevations of IP-10 and Mig in the mixed granulocyte subtype.

In the present study, asthma was divided into 3 phenotypes based on the predominant granulocyte in sputum. This kind of classification is generally considered clinically meaningful. This could lead to distinguishing features from the mixed granulocyte phenotype. Severe asthma is often characterized as airway inflammatory disease combined with elevated eosinophils and neutrophils [5, 6, 8, 13, 16, 21–23, 43]. The recent SARP study established this by showing that the rate of the mixed granulocyte phenotype was higher in severe asthma (45 %) [21]. The other analysis of a large number of subjects from the SARP study also showed that the mixed granulocyte phenotype had the poorest lung function and poor asthma control [23]. The present findings support the previous evidence. In the present study, the mixed granulocyte phenotype and the eosinophil or neutrophil-predominant subtype accounted for more than one quarter of severe asthma cases, and lung function in patients of this phenotype tended to be worse than that with the paucigranulocytic subtype. Airway inflammation in asthma is originally composed of the broad spectrum of diverse inflammatory cells. Many patients with asthma seem to exist at an intermediate stage between the paucigranulocyte phenotype and the other distinct granulocytic phenotypes. Taken together, this pathological entity may express the characteristics of asthma in real world practice.

There are some limitations in this study. First, this was a small scale study. This may affect the results in terms of the values of chemokines based on disease severity. Second, CXC chemokines were not evaluated by dividing asthma into the eosinophilic-predominant phenotype and the neutrophilic-predominant phenotype. To further interpret the present findings, further study may be needed. Third, this is a cross sectional study design.

## Conclusion

This study showed that IP-10 and Mig as well as IL-8 elevated in an asthma phenotype with increase of eosinophils combined with neutrophils in airway. These CXCR3 ligands were also positively correlated with the total ratio of granulocytes in sputum, which seems relevant to fixed airway obstruction. Mig was also associated with the decline of pulmonary function. Consequently, CXCR3 ligands, especially Mig, may be implicated in the combination of Th2-type and non-Th2-type bronchial inflammation in asthma.

## Additional files

Additional file 1: Table S1. Characteristics of subjects classified by clinical severity according to International ERS/ATS guidelines. (XLSX 12 kb)
Additional file 2: Table S2. Sputum Cell characteristics, and CXC3 ligands and IL-8 in subjects classified by clinical severity. (XLSX 11 kb)

## Abbreviations

ANOVA: A repeated-measures analysis of variance
ATS: American Thoracic Society
BAL: Bronchoalvelor lavage
CI: Confidence interval
CS: corticosteroid
ELISA: Enzyme-linked immunosorbent assay
ERS: European Respiratory Society
FeNO: Fraction of exhaled nitric oxide
FEV_1_: Forced expiratory volume in 1 s
FVC: forced vital capacity
GINA: Global Initiative For Asthma
HBSS: Hanks’ balanced salt solution
ICS: Inhaled corticosteroid
IFN: Interferon
IL: Interleukin
IP-10: FN-γ–inducible protein 10 kDa
I-TAC: IFN-inducible T cell a chemoattractant
LTRA: Leukotriene receptor antagonist.
Mig: monokine induced by IFN-γ
OCS: Oral corticosteroid
ppb: parts per billion
SARP: Severe Asthma Research Program
SEM: Standard error of the mean
